# The association of metabolic syndrome and cognitive impairment in Jidong of China: a cross-sectional study

**DOI:** 10.1186/s12902-021-00705-w

**Published:** 2021-03-04

**Authors:** Xiaohui Wang, Long Ji, Zhaoyang Tang, Guoyong Ding, Xueyu Chen, Jian Lv, Yanru Chen, Dong Li

**Affiliations:** 1 Department of Epidemiology, School of Public Health, Shandong First Medical University & Shandong Academy of Medical Sciences, 619 Changcheng Road, Taian, 271016 P.R. China; 2 The Second Affiliated Hospital of Shandong First Medical University, 706 Taisan Street, Taian, 271000 China

**Keywords:** Metabolic syndrome, Cognitive impairment, Epidemiology, Abdominal obesity, Blood pressure

## Abstract

**Background:**

Metabolic syndrome (Mets) is prevalent in the general population and has been reported to be an independent risk factor for cognitive impairment. This study aimed to investigate the association of Mets with the risk of cognitive impairment.

**Methods:**

We studied 5854 participants from the *Jidong* community. Cognitive function was assessed by the Mini-Mental State of Examination (MMSE) scale. Mets was diagnosed according to the International Diabetes Federation criteria. We used logistic regression analysis to investigate the association of metabolic syndrome with the risk of cognitive impairment.

**Result:**

Among the 5854 adults included in the study, the age mean (SD) of age was 44 (13.57) years, and 2916 (50.34%) were male. There was a higher (56.03%) cognitive impairment incidence rate among participants with Mets than among those without Mets. In addition, there was a significant association between Mets and cognitive impairment (OR: 2.39, 95% CI: 2.00–2.86, *P* < 0.05) after adjusting for potential confounders, including age, gender, education level, marital status, smoking and alcohol consumption status. Regarding the 5 Mets components, abdominal obesity and elevated blood pressure were associated with the risk of Mets (OR: 1.36, 95% CI: 1.09–1.70, *P* < 0.001; OR: 1.32, 95% CI: 1.07–1.63, *P* < 0.05). Moreover, the strongest statistical correlation (adjusted OR: 1.86, 95% CI: 1.22–2.83, *P* < 0.05) was found when the number of Mets components was three.

**Conclusion:**

Our study suggested that Mets was associated with cognitive impairment and that abdominal obesity and hypertension were associated with an increased risk of cognitive impairment.

## Background

At present, population aging has become a serious problem in many parts of the world. A large amount of data have indicated that the proportion of older people will increase to 31% in 2050, and China will have the greatest number of older people worldwide [1]. More seriously, related diseases such as cardiovascular disease and cognitive dysfunction related to aging also significantly reduce quality of life and increase the medical burden among the elderly [2].

Cognitive impairment is a well-known disease characterized by a reduction in cognitive function beyond what was expected from normal aging. Cognitive impairment involves functions in many areas of the brain, including areas associated with memory, thinking, orientation, comprehension, calculation, learning capacity, language, judgment and daily activities [3]. Epidemiological studies have indicated that the prevalence of mild cognitive impairment (MCI) varies from 2.8–17.5% in Europe and North America and 5.4–25.0% in different parts of China [4].

Some clinical and epidemiological studies have suggested that metabolic syndrome (Mets) plays an important role in the progression of cognitive impairment [5, 6]. Mets is a combination of cardiovascular risk factors (abdominal obesity, dyslipidemia, hyperglycemia, and hypertension) [7]. Mets is prevalent among adults worldwide. For example, the prevalence of Mets among urban adults from 33 communities in China was 27.4% [8], and the age-adjusted prevalence of Mets was 23% in the US general population and 30.52% in South Korea [9, 10].

Over the last few years, extensive research and multiple reviews have suggested that there is a link between Mets and cognitive impairment [11]. In the Sacramento Area Latino Study of Aging Study, it was reported that Mets contributes to cognitive decline, and the composite measure of Mets is associated with higher odds than individual components [12]. A recent study using a rat model of Mets found that high fructose intake resulted in disrupted insulin signaling in the brain [13]. However, previous studies reported that there was no association between Mets and cognitive impairment among older US adults [14]. In addition, a previous longitudinal study showed that metabolic syndrome was a protective factor for cognitive function decline [12, 15, 16]. The inconsistent results may be due to differences in age, education and other confounders.

Therefore, the primary aim of our study was to explore whether Mets was associated with the risk of cognitive impairment.

## Methods

### Study population

The cross-sectional study was based on the China suboptimal health cohort study (COACS), a longitudinal study initiated in 2013. We recruited 6653 participants from *Tangshan*, Hebei Province, in northern China in 2015. In addition, 799 participants were excluded due to incomplete baseline information and Mini-Mental State of Examination (MMSE) scores. Finally, 5854 individuals were included in our present study. The study was approved by the ethics committee of *Jidong* Oilfield Inc. [17] All participants received adequate information about the study and provided written informed consent.

### Physical examination and assessment of metabolic syndrome

Diastolic blood pressure (DBP) and systolic blood pressure (SBP) were measured three times using a standard mercury sphygmomanometer by well-trained nurses. Waist circumference was measured in centimeters at the midpoint between the lowest rib margin and the top of the iliac crest at minimal respiration to the closest 0.1 cm. Fasting plasma glucose (FPG) was measured with the hexokinase/glucose-6-phosphate dehydrogenase method. Triglycerides (TGs) were determined by enzymatic methods (Mind Bioengineering Co. Ltd. Shanghai, China). High-density lipoprotein cholesterol (HDL-C) was measured using an autoanalyzer (Hitachi 747; Hitachi, Tokyo, Japan) at the abdominal laboratory of the Staff Hospital of *Jidong* oilfield of Chinese National Petroleum [17].

The revised criteria of the International Diabetes Federation (IDF) criteria was used to define Mets [18]. The criteria emphasize central obesity as essential condition, that was assessed by waist circumference (≥ 90 cm for Chinese men and ≥ 80 cm for Chinese women). Besides, the diagnostic criteria of Mets need to plus at least 2 the following cardiovascular risk factors (CVRFs). 1) raised triglycerides: ≥ 150 mg/dl (1.7 mmol/l) or specific treatment for this lipid abnormality. 2) reduced HDL-cholesterol: < 40 mg/dL (1.03 mmol/l) in men and < 50 mg/dL (1.29 mmol/l) in women or specific treatment for this lipid abnormality. 3) raised blood pressure: systolic ≥130 mmHg or diastolic ≥85 mmHg or treatment of previously diagnosed hypertension. 4) raised fasting plasma glucose: fasting plasma glucose ≥100 mg/dL (5.6 mmol/l) or previously diagnosed type 2 diabetes.

### Cognitive measures and other covariates

The MMSE was used to assess the participants’ cognitive function. The MMSE consists of 30 items assessing memory, attention, language, calculation, visuospatial abilities and orientation [19]. The score ranges from 0 to 30, and higher scores represent better cognition. In prior studies, it had been reported that the cutoff is 27 for individuals with more than 7 years of literacy [20, 21]. The MMSE 27 cutoff had a higher sensitivity (94.9%) and specificity (66.3%) than the MMSE 24 cutoff [22]. Therefore, cognitive impairment was defined as a score less than 27 in our study.

Clinical characteristics and biochemical indicators were collected by clinical and laboratory tests. Questionnaires were used to collect information related to demographic variables and behavioral lifestyle [17]. The covariates included gender, age, education level, marital status, smoking and alcohol consumption status, WC, serum TGs, HDL-C, SBP, DBP, and FPG.

### Statistical analysis

**Fig. 1:**
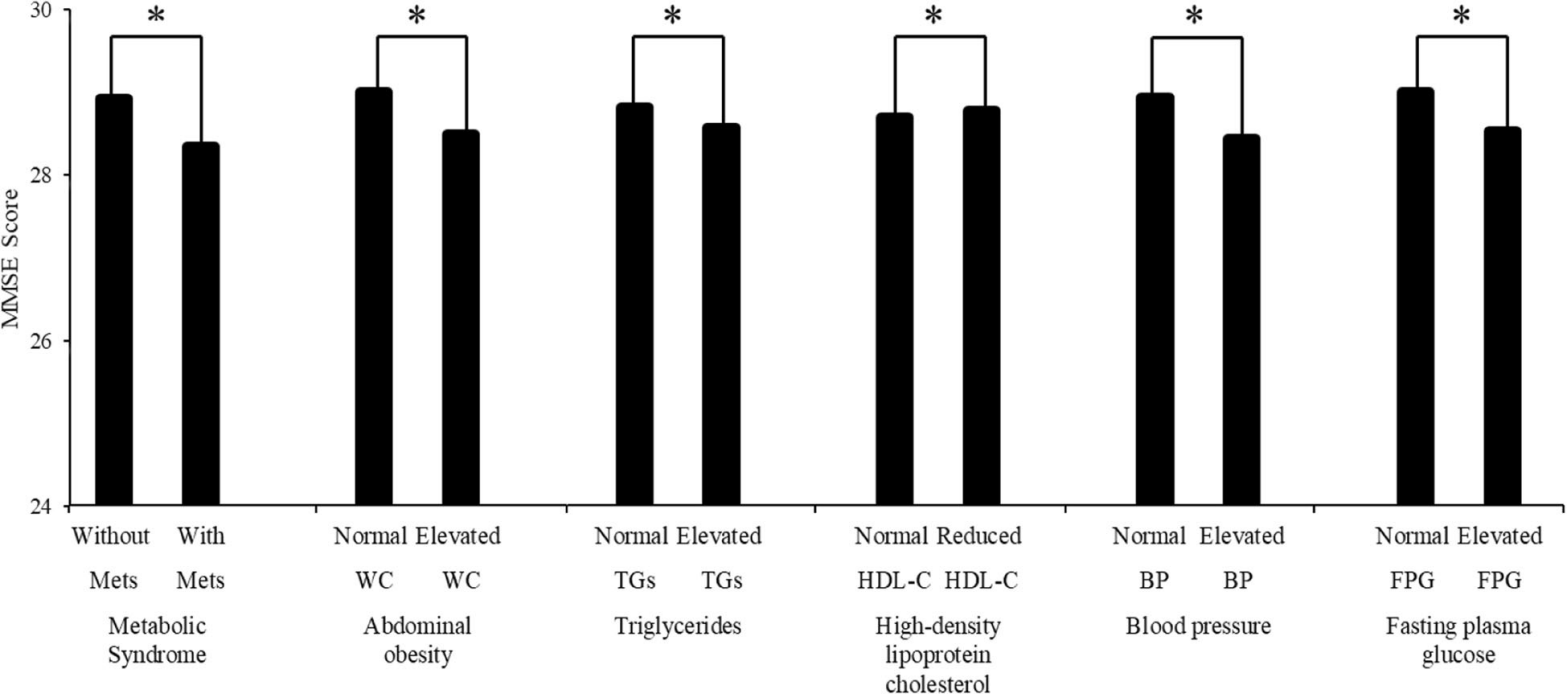
Comparisons of MMSE scores between those with and without metabolic syndrome and its components. MMSE: Mini-Mental State of Examination, Mets: metabolic syndrome, Non-Mets: non-metabolic syndrome, BMI: body mass index, WC: waist circumference, TGs: triglycerides, HDL-C: high-density lipoprotein cholesterol, SBP: systolic blood pressure, DBP: diastolic blood pressure, FPG: fasting plasma glucose, WC: waist circumference, *: *p* ≤ 0.05

For baseline characteristics, the Kolmogorov-Smirnov test was used to evaluate the normal distribution of continuous variables. The continuous variables with normal distribution are expressed as the mean ± standard deviation, and the categorical variables are presented as numbers (percentages). Then, continuous variables with normal distribution were compared using Student’s t-test, and categorical variables were compared using chi-square test analysis. Nonnormally distributed variables were compared using nonparametric (Table 1). Next, the individuals were divided into normal and elevated or reduced groups according to the diagnostic criteria of Mets, and the t-test was used to compare the differences in MMSE scores between the two groups (Fig. 1).

**Table 1:** Baseline characteristics of the study population according to metabolic syndrome

Characteristics	Total	MetS	Non-MetS	*P*
Number of subjects (n, %)	5854	2154 (36.80)	3700 (63.20)	
Age (years), mean ± SD	43.98 ± 13.58	48.95 ± 13.61	41.07 ± 12.69	< 0.001*
Male (n, %)	2944	1222 (56.73)	1722 (46.55)	< 0.001*
Education level (n, %)				< 0.001*
Illiteracy/primary school	357	205 (9.52)	152 (4.11)	
Middle school	2013	951 (44.15)	1062 (28.70)	
College or above	3484	998 (46.33)	2486 (67.19)	
Marriage (n, %)				< 0.001*
Single	409	103 (4.78)	306 (8.27)	
Married	5445	2051 (95.22)	3394 (91.73)	
Current smoking (n, %)	1542	712 (33.05)	830 (22.43)	< 0.001*
Current alcohol (n, %)	1852	784 (36.40)	1068 (28.86)	< 0.001*
BMI (kg/m^2^), median (interquartile range)	24.3 (21.9, 26.8)	27.00 (25.1,29.1)	22.8 (20.9,24.7)	< 0.001*
WC (cm), median (interquartile range)	86 (78,93)	94 (90,100)	81 (75,87)	< 0.001*
TGs (mmol/L), median (interquartile range)	1.48 (1.04,2.24)	2.19 (1.61,3.06)	1.21 (0.92,1.62)	< 0.001*
HDL-C (mmol/L), median (interquartile range)	1.22 (1.06,1.40)	1.12 (0.98,1.28)	1.27 (1.12,1.46)	< 0.001*
SBP (mmHg), median (interquartile range)	125 (113,136)	136 (127,148)	118 (110,128)	< 0.001*
DBP (mmHg), median (interquartile range)	79 (71,88)	87 (79,95)	75 (68,82)	< 0.001*
FPG (mmol/L), median (interquartile range)	5.72 (5.42,6.09)	6 (5.72,6.57)	5.56 (5.32,5.86)	< 0.001*
Elevated TGs (n, %)	2354	1556 (72.24)	798 (21.57)	< 0.001*
Low HDL-C (n, %)	879	565 (26.23)	314 (8.49)	< 0.001*
Elevated BP (n, %)	2648	1686 (78.27)	962 (26.00)	< 0.001*
Elevated FPG (n, %)	3572	1848 (85.79)	306 (46.59)	< 0.001*
MMSE, mean ± SD	28.71 ± 1.81	28.35 ± 2.10	28.93 ± 1.57	< 0.001*
MMSE< 27(n, %)	544	306 (14.21)	238 (6.43)	< 0.001*

*sMets* Metabolic syndrome, *Non-Mets* Non-metabolic syndrome, *BMI* Body mass index, *WC* Waist circumference, *TGs* Triglycerides, *HDL-C* High-density lipoprotein cholesterol, *SBP* Systolic blood pressure, *DBP* Diastolic blood pressure, *FPG* Fasting plasma glucose, *MMSE* Mini-mental state of examination, *: *P* ≤ 0.05: significant difference from Mets and Non-Mets

Univariate and multivariate logistic regression were used to assess the association between Mets and cognitive impairment. Moreover, we used three regression models in the analysis: Model 1 was an unadjusted model, Model 2 was adjusted for age, gender, education, marital status, current smoking, and current alcohol consumption; Model 3 additionally included abdominal obesity, elevated TG, reduced HDL-C, elevated BP and elevated FPG.

Next, we inputted the number of Mets components into multivariate logistic regression to evaluate the effects of the number of abnormal Mets components on cognitive impairment. Two models were generated: one made no adjustments, and the other controlled for age, gender, education, marital status, current smoking, and current alcohol consumption.

All statistical analyses were performed by SAS software, version 9.4 (SAS Institute Inc., Cary, North Carolina, USA). A *P*-value< 0.05 was considered statistically significant.

## Results

Table 1 summarizes the demographic data of 5854 participants. There were 2154 (36.80%) participants with Mets and 3700 (63.20%) participants without Mets. Of the 2154 participants with Mets, 1556 (72.24%) had hypertriglyceridemia, 565 (26.23%) had low HDL-C, 1686 (78.27%) had high blood pressure or were diagnosed with hypertension, and 1848 (85.79%) had high FPG or were diagnosed with hyperglycemia. The age mean ± standard deviation of the people with Mets was 43.98 ± 13.58. The current study showed that older married men with smoking and alcohol abuse habits were more likely to have Mets than those without these habits (*P* < 0.05). The participants who were better educated had a lower prevalence of Mets (*P* < 0.05).

Comparisons of MMSE scores between individuals with Mets and its four components are illustrated in Fig. 1. The MMSE scores mean ± standard deviation (28.35 ± 2.10) of participants with Mets was significantly lower than that participants without Mets (28.93 ± 1.57). People with normal WC, TGs, BP and FPG had higher MMSE scores than those with elevated WC, TGs, BP, FPG, people with normal HDL-C had lower MMSE scores than that with reduced HDL-C. All differences were statistically significant (*P* < 0.05). (The concepts of normal and elevated or reduced are consistent with the diagnostic criteria of Mets.

**Figure 2:**
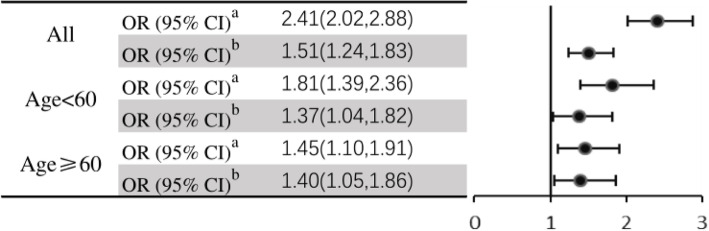
The odds of cognitive impairment according to metabolic syndrome in different age groups. OR, odds ratio. ^a^: no adjustment, ^b^: adjustment age, gender, education, marital status, current smoking, current drinking

We further investigated the risk of cognitive impairment and Mets in different age groups (60 years) in Fig. 2. Participants with Mets had 2.41-fold odds of having cognitive impairment in the crude model (OR 2.41, 95% CI: 2.01–2.88, *P* < 0.001), and the association was consistent when controlling for gender, current smoking, and current alcohol consumption (OR 1.51, 95% CI: 1.24–1.83, *P* < 0.001). After the stratified analysis, the association between Mets and cognitive impairment remained significant. In the group aged < 60 years, the unadjusted and adjusted odds ratios (ORs) and 95% CIs were 1.812 (1.39, 2.36) and 1.374 (1.04, 1.82), respectively. In the other group (age ≥ 60 years), the unadjusted and adjusted ORs and 95% CIs were 1.45 (1.10, 1.91) and 1.40 (1.05, 1.86), respectively.

**Fig. 3:**
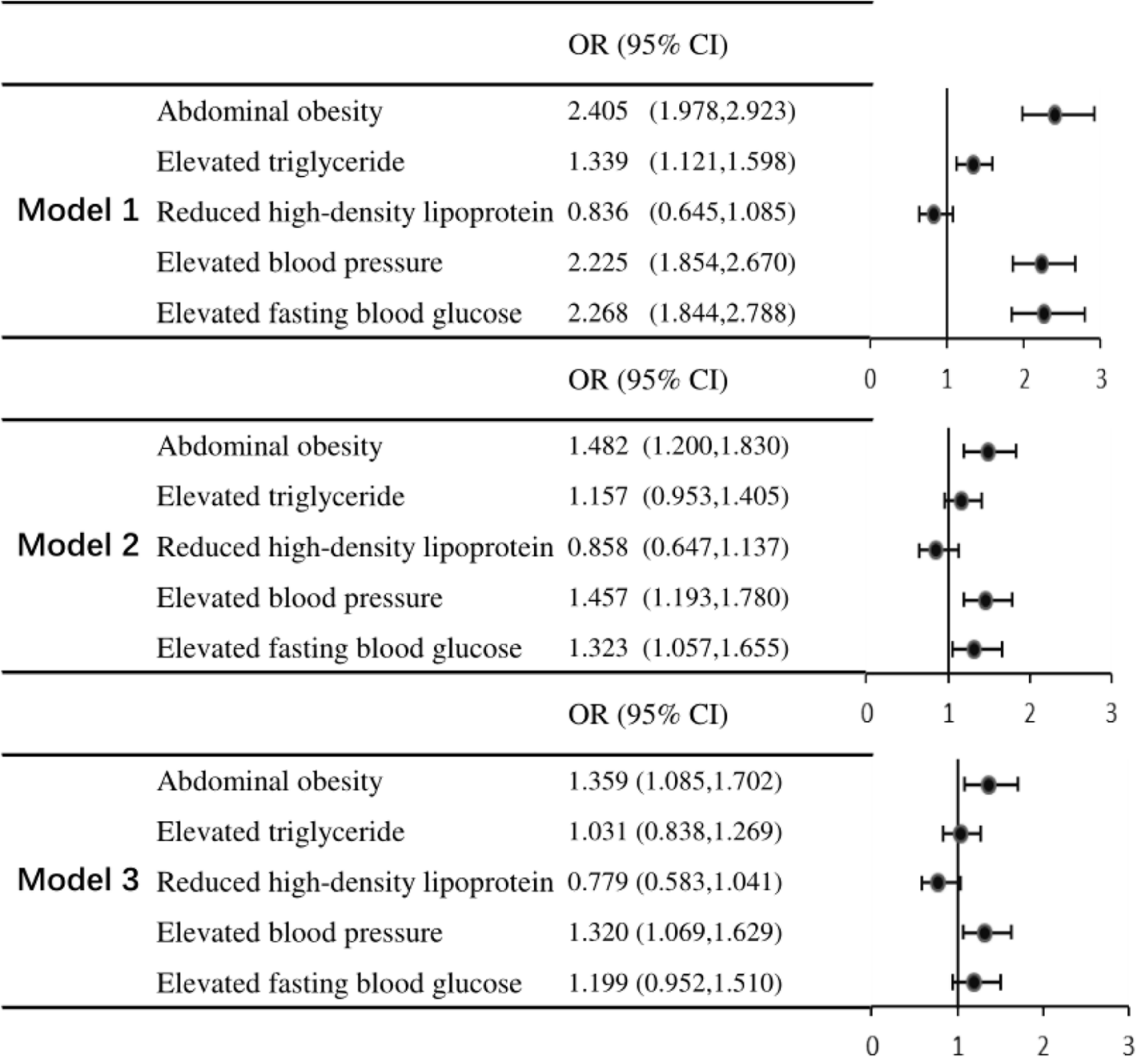
Mets, each of the 5 Mets components and odds of cognitive impairment**.** Mets: metabolic syndrome, Non-Mets: Non-metabolic syndrome, CI, confidence interval, OR, odds ratio. Model 1: no adjustment, Model 2: adjustment age, gender, education, marital status, current smoking, current drinking, Model 3: model 2 plus abdominal obesity, elevated triglycerides, reduced high-density lipoprotein, elevated blood pressure, elevated glucose

Figure 3 provides information on Mets, each of the 5 Mets components and the odds of cognitive impairment**.** Abdominal obesity, elevated triglyceride, elevated blood pressure, and elevated blood glucose were found to be significantly associated with cognitive impairment in Model 1 (all *P* < 0.001; Fig. 3), but this association was not seen for low high-density lipoprotein.

In Model 2, abdominal obesity had an OR of 1.48 (95% CI: 1.20–1.83, *P* < 0.001), elevated blood pressure had an OR of 1.46 (95% CI: 1.19–1.78, *P* < 0.001), and elevated blood glucose had an OR of 1.32 (95% CI: 1.06–1.66, *P* = 0.014). However, elevated triglyceride and reduced high-density lipoprotein were not associated with cognitive impairment (all *P* > 0.05, Fig. 3).

In Model 3, abdominal obesity and elevated blood pressure both had statistically significant results (OR 1.36, 95% CI: 1.09–1.70, *P* = 0.007; OR 1.32, 95% CI 1.07–1.63, *P* = 0.010). However, elevated triglyceride, reduced high-density lipoprotein and elevated blood glucose were not significantly associated with cognitive impairment (all *P* > 0.05, Fig. 3).

**Fig. 4:**
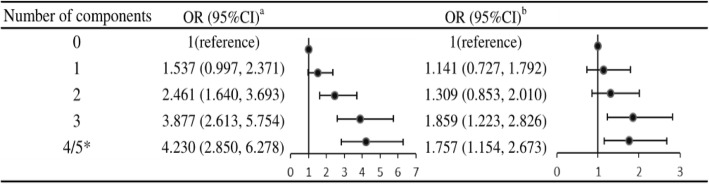
Number of Mets components and odds of cognitive impairment**.** Mets: metabolic syndrome, Non-Mets: Non-metabolic syndrome, ^a^:no adjustment, ^b^: adjusted for age, gender, education, marital status, current smoking, current drinking, CI, confident interval, OR, odds ratio, * due to small in each, group with 4 or 5 components were merged in this analysis

The number of Mets components was related to cognitive impairment. Compared to the reference group with 0 components, the adjusted ORs and 95% CI for subjects in the groups with 3 and 4/5 Mets components were 1.86 (1.22–2.83) and 1.76 (1.15–2.67), respectively. However, similar results were not found in the group with 1 and 2 Mets components (*P* > 0.05; Fig. 4).

## Discussion

In this community-based cross-sectional study, we found that the participants with Mets had lower MMSE scores. In addition, our results still showed a correlation between Mets and cognitive impairment after stratification by age. In this study, we observed that abdominal obesity and hypertension were independent risk factors for cognitive impairment. We also found that higher levels of education were associated with better cognitive functioning and that older age and married status were associated with worse cognitive impairment. Furthermore, we explored the relationship between Mets and cognitive impairment, and the data suggested that the strongest risk was associated with the presence of three Mets components.

According to the present study, the correlation between Mets and cognitive function is still unclear, and the conclusions are not completely consistent. Previous studies have shown that hypertension and hyperglycemia may be linked to cognitive function [23]. Age and abdominal obesity were significant risk factors for cognitive decline [16]. Studies have failed to show any association between Mets and cognitive impairment [14, 24]; however, most studies have shown that participants with metabolic syndrome are associated with increased odds of cognitive impairment [12, 15, 19, 23, 25],which is consistent with our results. Although most studies have shown that Mets and its components play roles in cognitive decline, other studies have suggested that late-life Mets has a protective effect on cognitive function [16] .

Our findings provide evidence that with the presence of abdominal obesity predicts a higher risk of cognitive impairment. The significant association was still present even after multivariable adjustments. Then, the effects of overweight on brain function may be achieved through several mechanisms. First, abdominal obesity had a stronger association with visceral adiposity than body mass index (BMI) [26] . The accumulation of visceral fat leads to metabolic disorders. Adipocytes absorb glucose, damage insulin receptor basal protein insulin signal reception, and induce insulin resistance, and insulin resistance has been defined as a potential modifiable risk factor for Alzheimer’s disease (AD) [27, 28] .Second, overweight reduces serum adiponectin (APN) concentration. A lack of APN may lead to loss of neurons and synapses in the brain, increased brain Aβ-42 levels, deposition of amyloid-β protein, and increased microglia and astroglia, leading to cognitive impairment [29–31] .

The current findings were consistent with previous work that demonstrated a relationship between chronic hypertension and reduced cognition. The effects of hypertension on cognitive function were mainly achieved through the following aspects.

On the one hand, hypertension leads to arterial smooth muscle hyperplasia, vascular remodeling, and the formation of atherosclerosis, which may promote reactive oxygen species production and inflammation in cerebral blood vessels. Oxidative stress and the inflammatory response are important mechanisms of cognitive impairment. On the other hand, hypertension destroys the mechanism of cerebral blood flow regulation, which compromises the clearance of brain metabolites, such as amyloid-β and tau, favoring their accumulation. The accumulation of amyloid-β and tau are also important mechanisms of cognitive impairment.

Previous studies have reported that type 2 diabetes mellitus (T2DM) is a risk factor for AD, and an important mechanism may be changes in brain insulin levels [32, 33]. However, a difference between hyperglycemia and cognitive impairment was not found in our study. The percentage of participants with normal blood glucose levels was 61.02%, which may partially explain why we did not observe a significant association between blood glucose and cognitive impairment. However, as shown in Fig. 2, there was a significant increase (OR = 1.199) in the risk of cognitive impairment in people with hyperglycemia.

This study had limitations. First, this study used a cross-sectional design, which allowed us to explore a cause-and-effect relationship. Second, the sample may not have been representative because the participants have a high education level. Third, we were unable to scientifically assess cognitive impairment. We measured cognitive impairment with only the MMSE, which is not a professional neurocognitive assessment. Moreover, we used 27 as the MMSE cutoff-off point rather than 24 considering the high level of education in the *Jidong* community.

## Conclusion

In this community-based cross-sectional study, Mets was associated with the risk of cognitive impairment, and the difference was still significant in age subgroups. Our study supported abdominal obesity and hypertension as independent risk factors for cognitive impairment. Mets was associated with cognitive impairment, and preventing Mets and its components may reduce the incidence of cognitive impairment. Prospective studies on more diverse populations and the causal role of Mets in the development of cognitive impairment are needed.

## Data Availability

The data used to support the findings of this study are available from the corresponding auth or upon request.

## Abbreviations

Mets: Metabolic syndrome
MMSE: Mini-mental state of examination
SD: Standard deviation
OR: Odds ratio
CI: Confidence interval
MCI: Mild cognitive impairment
US: United States
COACS: China suboptimal health cohort study
DBP: Diastolic blood pressure
SBP: Systolic blood pressure
FPG: Fasting plasma glucose
TGs: Triglycerides
HDL-C: High-density lipoprotein cholesterol
CVRFs: Cardiovascular risk factors
BMI: Body mass index
AD: Alzheimer’s disease
APN: Adiponectin
T2DM: Type 2 diabetes mellitus
